# Topological edge properties of C_60+12_*_n_* fullerenes

**DOI:** 10.3762/bjnano.4.47

**Published:** 2013-06-26

**Authors:** A Mottaghi, Ali R Ashrafi

**Affiliations:** 1 Department of Nanocomputing, Institute of Nanoscience and Nanotechnology, University of Kashan, Kashan 87317-51167, I R Iran

**Keywords:** edge revised Szeged index, edge Szeged index, edge topological property, fullerene, PI index

## Abstract

A molecular graph M is a simple graph in which atoms and chemical bonds are the vertices and edges of M, respectively. The molecular graph M is called a fullerene graph, if M is the molecular graph of a fullerene molecule. It is well-known that such molecules exist for even integers *n* ≥ 24 or *n* = 20. The aim of this paper is to investigate the topological properties of a class of fullerene molecules containing 60 + 12*n* carbon atoms.

## Introduction

Throughout this paper the term "graph" refers to a finite and simple graph. The set of vertices and edges of a graph *G* are denoted by *V*(*G*) and *E*(*G*), respectively. Molecular graphs are graphs with vertices representing the atoms and edges representing the bonds. A bi-connected graph is a connected graph in which, by removing any vertex, the graph will remain connected. A graph in which all vertices have degree three is called a cubic graph. A fullerene graph is a cubic bi-connected planar graph whose faces are pentagons and hexagons. From Euler’s theorem, one can easily see that such graphs have exactly 12 pentagonal and (*n*/2 − 10) hexagonal faces, where 20 ≤ *n.* It is not so difficult to prove that there is no fullerene with exactly 22 carbon atoms. After the discovery of buckminsterfullerene C_60_ by Kroto and Smalley in 1985 [1–2], some mathematicians spent their time looking at the mathematical properties of these new materials. We refer to [3] for more information on the mathematical properties of fullerene graphs.

Suppose *G* is a graph. A mapping *f*: *G* → *G* is an automorphism if and only if (i) *f* is one-to-one and (ii) *f* and its inverse preserve adjacency in *G*. The property *P* on *G* is called a topological property if *P* is preserved under each automorphism of *G.* A topological index is a number describing a topological property. It should be applicable in chemistry. The length of a shortest path connecting vertices *u* and *v* is called the topological distance between *u* and *v*, denoted by *d*(*u,v*). A topological index is considered to be distance-based, if it can be defined by a function *d*.

Suppose *e = uv* 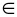 *E*(*G*). Then *m_u_*(*e*) is defined as the number of edges closer to *u* than *v* and *m_v_*(*e*) can be defined in a similar way. The PI and edge Szeged indices are basis dual indices defined as *PI*(*G*) = ∑*_e=uv_*[*m_u_*(*e*) *+ m_v_*(*e*)] and *Sz_e_*(*G*) = ∑*_e=uv_m_u_*(*e*)*m_v_*(*e*), respectively. Interested readers can consult [4–6] and references therein for more information on these graph invariants. A modification of the Szeged index was proposed by Milan Randić in [7]. Some mathematical properties of this topological index are investigated in [8–9]. One of the present authors (ARA) [10] proposed an edge version of the revised Szeged index as follows:


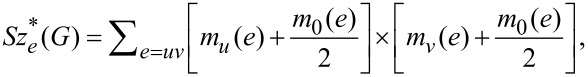


where *m*_0_(*e*) denotes the number of vertices equidistant from *u* and *v*. In [11], some mathematical properties of this new graph invariant have been investigated.

**Figure 1:**
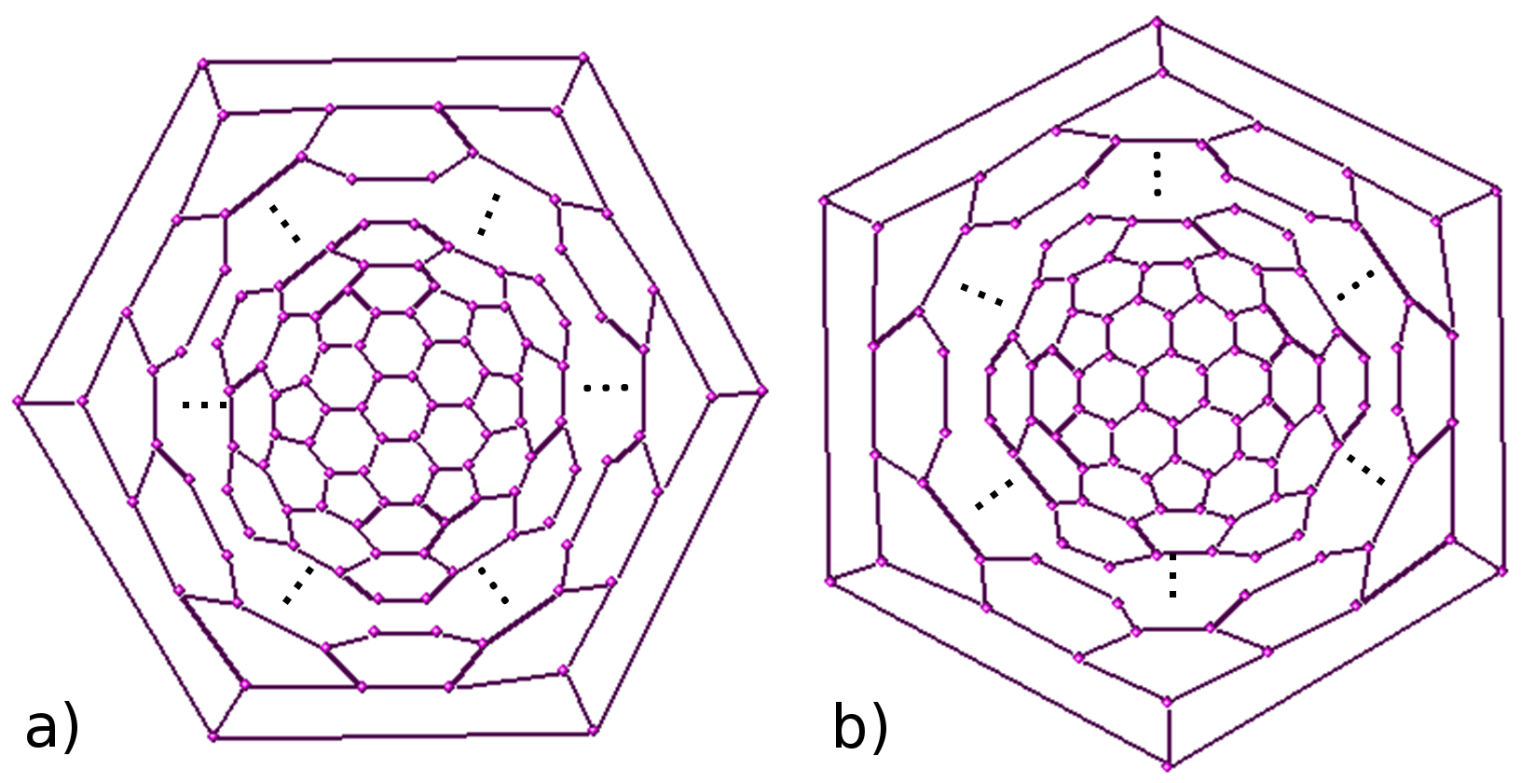
The Schlegel diagram of *F_n_*, (a) *n* is even; (b) *n* is odd.

The aim of this paper is to compute PI, edge Szeged and edge revised Szeged indices of an infinite class *F_n_* of fullerenes with exactly 60 + 12*n* carbon atoms (Figure 1). We encourage the interested readers to consult [12–14] for some extraordinary works on this topic and [15–19] for background materials and basic computational techniques. Our calculations are done with the aid of HyperChem [20], TopoCluj [21] and GAP [22]. Our notation is according to the standard books on graph theory.

## Results and Discussion

Khadikar and co-authors [4] were the first scientists to consider the topological edge properties of molecules. In this section, we will compute the PI, edge Szeged and edge revised Szeged indices of *F_n_*. We can associate a 0–1 matrix *A* = [*a_ij_*] to *F_n_*. The entry *a_ij_* is unity if and only if the vertices *i* and *j* are adjacent in *F_n_*. Since *F_n_* is a cubic cage graph, the number of units in each row of *A* is equal to 3. The distance matrix *D* = [*d_ij_*] is another *n* × *n* matrix associated to *F_n_*. Here, *d_ij_* is the length of a minimal path connecting *i* and *j*, for *i* ≠ *j*, and zero otherwise. Our algorithm for computing the PI, edge Szeged and edge revised Szeged indices of the fullerene graph *F_n_* is as follows: We first draw the fullerene by HyperChem. Then we upload the hin file of the fullerene into TopoCluj. By computing the adjacency and distance matrices of *F_n_* by TopoCluj, we calculate the PI, Szeged and revised Szeged indices of our fullerene graph by some GAP programs. These computer programs are accessible from the authors upon request.

**Figure 2:**
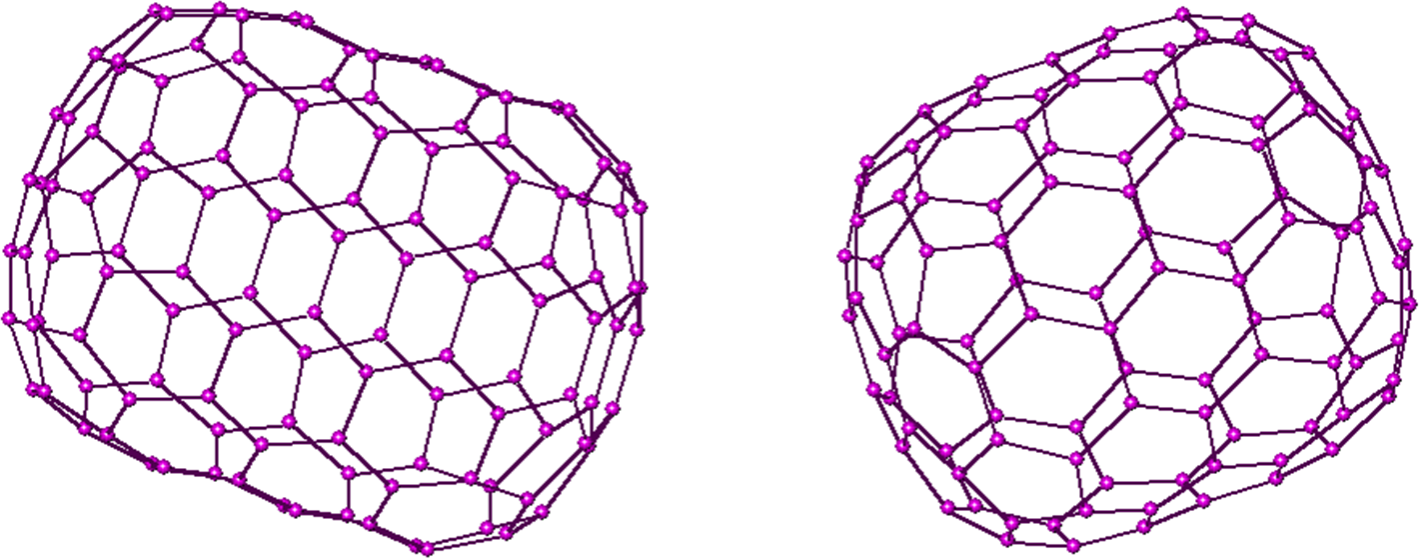
The 3D view of fullerenes C_108_ (left) and C_96_ (right).

**Figure 3:**
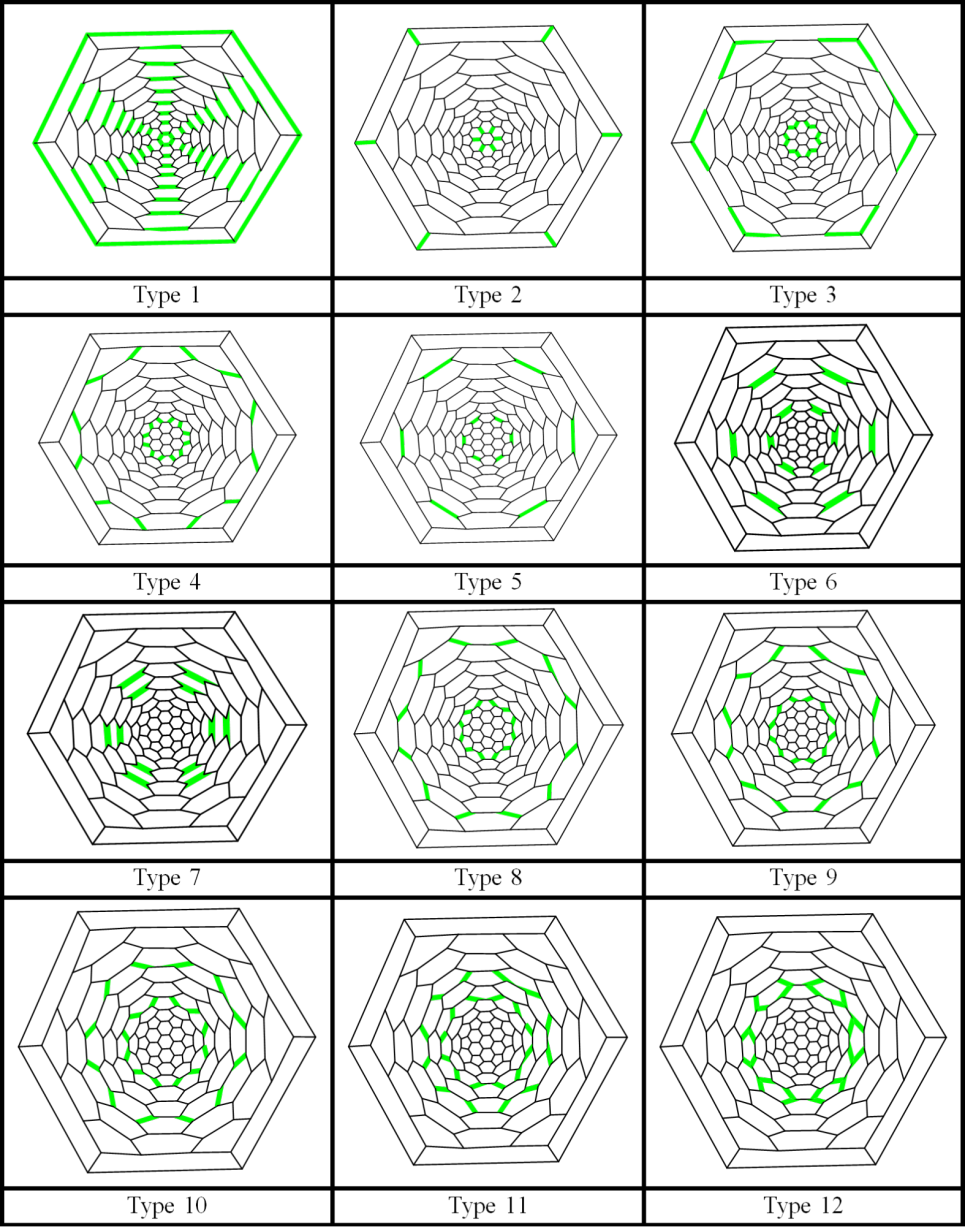
Twelve different types of edges of *F_n_*, *n* is even.

**Figure 4:**
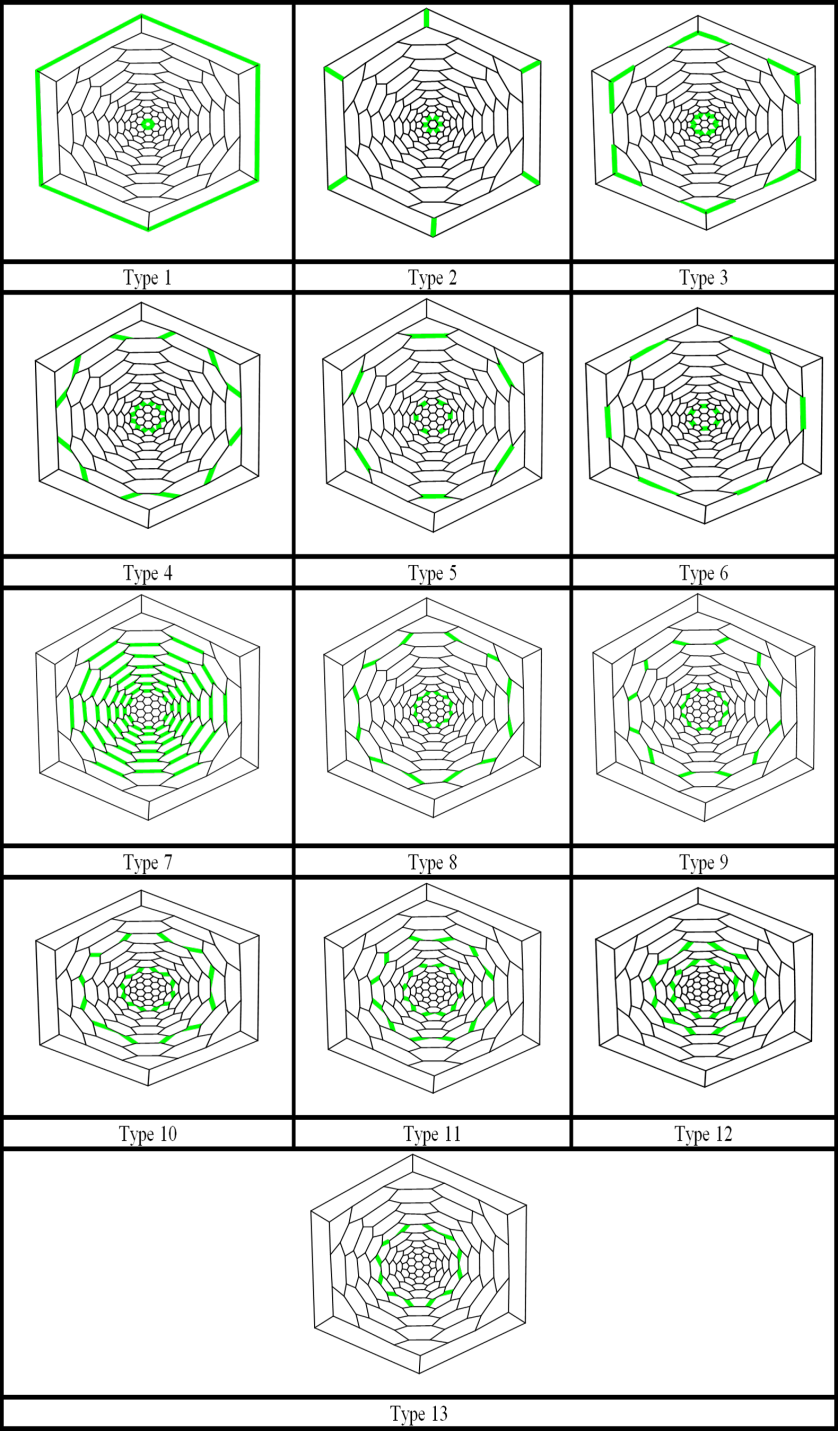
Thirteen different types of edges in *F_n_*, *n* is odd.

In Figure 1 and Figure 2, the 2D and 3D perception of *F_n_* are depicted. We apply our mentioned method for some small numbers of *n*. Using our programs, we obtain seven exceptional cases, those of *n =* 1 to 7. In Table 1, the quantities *m_u_*(*e*), *m_v_*(*e*) and *m*_0_(*e*) = 90 *+* 18*n – m_u_*(*e*) – *m_v_*(*e*) for these exceptional cases are recorded. We notice that there are two cases, that is, when *n* is odd or even. If *n* is even then we have 12 different types of edges, Figure 3, and if *n* is odd then there are 13 different types of edges (Figure 4).

**Table 1:** The values of *m_u_*(*e*), *m_v_*(*e*) and *m_0_*(*e*) for different types of edges, when *n* ≤ 7.

C_72_	C_84_	C_96_	C_108_	C_120_	C_132_	C_144_	Number of edges

49,49,10	—	66,66,12	—	83,83,14	—	100,100,16	12
—	58,58,10	—	—	—	—	—	30
—	—	—	75,75,12	—	—	—	36
—	—	—	—	—	92,92,14	—	42
36,51,21	43,57,26	49,62,33	56,68,38	62,73,45	69,79,50	75,84,57	12
37,48,23	49,51,26	55,53,36	68,53,41	74,53,53	87,53,58	93,53,70	24
45,45,18	—	—	—	—	—	—	12
—	50,53,23	—	—	—	—	—	48
—	—	—	—	80,80,20	—	97,97,22	36
—	—	63,63,18	—	—	—	—	24
40,46,22	—	64,57,23	80,58,24	98,58,24	116,58,24	134,58,24	24
40,40,28	44,44,38	57,57,30	62,62,38	74,74,32	79,79,40	91,91,34	12
—	—	57,63,24	77,61,24	93,62,25	111,62,25	129,62,25	24
—	—	—	70,73,19	87,74,19	103,75,20	121,75,20	24
42,42,24	—	62,62,20	—	79,79,22	84,84,30	96,96,24	12
—	—	—	66,66,30	—	—	—	6
—	—	—	—	—	94,84,20	110,85,21	24
—	—	—	—	—	—	98,98,20	12

In Table 2, the quantities, PI, edge Szeged and edge revised Szeged indices for the exceptional cases 1 ≤ *n* ≤ 7 are recorded.

**Table 2:** The quantities, PI, edge Szeged and edge revised Szeged indices of *F_n_*, 1 ≤ *n* ≤ 7*.*

Index	*n* = 1	*n* = 2	*n* = 3	*n* = 4	*n* = 5	*n* = 6	*n* = 7

PI(*F_n_*)	9372	13080	—	—	—	—	—
Sz_e_(*F_n_*)	202296	340740	512796	753684	1031424	1392672	1798512
Sz_e_*(*F_n_*)	313311	499374	745455	1056606	1438611	1893468	2431545

In Table 3 and Table 4, the quantities of *m_u_*(*e*), *m_v_*(*e*) and *m_0_*(*e*) are calculated. By these tables and a case-by-case investigation on the molecular graph of *F_n_* led to the following observation: The PI, edge Szeged and edge revised Szeged indices of C_60_*_+_*_12_*_n_* fullerenes can be computed by the following formulae:


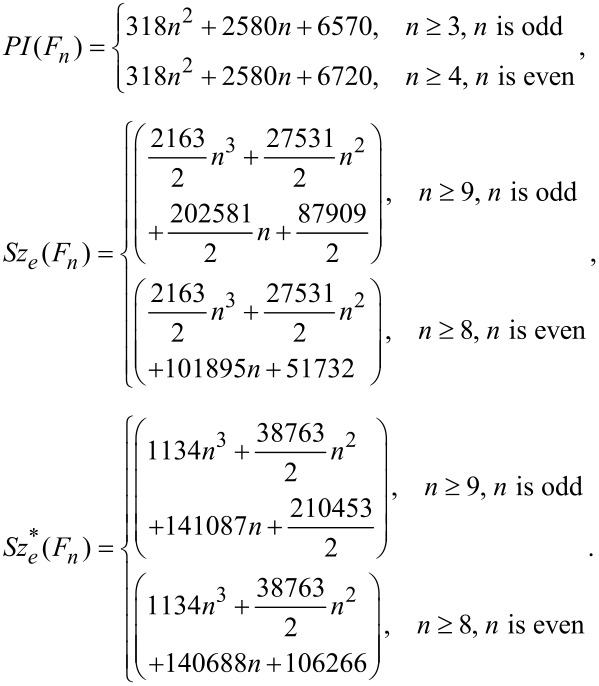


It is possible to find a proof for this observation by a tedious calculation on the molecular graph of *F_n_*.

**Table 3:** *m_u_*(*e*), *m_v_*(*e*) and *m_0_*(*e*) for different types of edges, *n* is odd and *n* ≥ 9.

Edges	*m_u_*(*e*), *m_v_*(*e*) and *m*_0_(*e*)	Number of edges

1	1/2(17*n* + 81), 1/2(17*n* + 81), *n*+9	12
2	1/2(13*n* + 59), 1/2(11*n* + 9), 6*n* + 15	12
*3*	1/2(19*n* + 53), 53, 1/2(17*n* + 21)	24
*4*	18*n* + 8, 58, 24	24
*5*	1/2(17*n* + 63), 1/2(17*n* + 63), *n* + 27	12
*6*	1/2(17*n* + 73), 1/2(17*n* + 73), *n* + 17	12
*7*	1/2(17*n* + 75), 1/2(17*n* + 75), *n* + 15	6(*n* − 1)
*8*	18*n* + 3, 62, 25	24
*9*	18*n* − 5, 75, 20	24
*10*	18*n* − 16, 85, 21	24
*11*	18*n* − 30, 99, 21	24
*12*	18(*n* − i) − 11, 18(4 + *i*) + 7, 22 *n* ≥ 6 + 2*i*, *i* = 2,3,…	24
*13*	9*n* + 34, 9*n* + 34, 22	12

**Table 4:** *m_u_*(*e*), *m_v_*(*e*) and *m_0_*(*e*) for different types of edges, *n* is even and *n* ≥ 8.

Edges	*m_u_*(*e*), *m_v_*(*e*) and *m*_0_(*e*)	The number of edges

1	1/2(17*n* + 82), 1/2(17*n* + 82), *n* + 8	3(*n* + 8)
2	1/2(13*n* + 60), 1/2(11*n* + 92), 6*n* + 14	12
3	1/2(19*n* + 60), 53, 1/2(17*n* + 14)	24
4	18*n* + 8, 58, 24	24
5	1/2(17*n* + 56), 1/2(17*n* + 56), *n* + 34	12
6	1/2(17*n* + 66), 1/2(17*n* + 66), *n* + 24	12
7	1/2(17*n* + 68), 1/2(17*n* + 68), *n* + 22	3(*n* − 6)
8	18*n* + 3, 62, 25	24
9	18*n* − 5, 75, 20	24
10	18*n* − 16, 85, 21	24
11	18*n* − 30, 99, 21	24
12	18(*n* – *i*) – 11, 18(4 + *i*) + 7, 22 *n* ≥ 6 + 2*i*, *i* = 2,3,…	24

## Conclusion

In this paper a computational method for computing PI, edge Szeged and edge revised Szeged indices of fullerene graphs is presented. In [18–19], the authors considered the topological properties of fullerenes given by vertex contributions of its molecular graph. In this work, the topological properties of a class of fullerenes were given by edge contributions of its molecular graph. Our calculations with this and other classes of fullerenes suggest that the edge PI index can be computed by a polynomial of degree 2, whereas edge Szeged and edge revised Szeged indices are computed by polynomials of degree 3. It is clear that we cannot characterize fullerenes by one topological index, but we can think about the possibility of characterizing these molecular graphs by a finite set, Ω, of topological indices. We guess that Ω contains at least two topological indices A and B, such that A and B can be computed by edge and vertex contributions, respectively.
